# Correlation between the single nucleotide polymorphisms of the human phosphodiesterase 4D gene and the risk of cerebral infarction in the Uygur and Han ethnic groups of Xinjiang, China

**DOI:** 10.3892/etm.2013.1370

**Published:** 2013-10-29

**Authors:** JIANHUA MA, QIMENG SUN, XIAONING ZHANG, HEBIN DU

**Affiliations:** Department of Neurology, The First Affiliated Hospital of Xinjiang Medical University, Urumqi, Xinjiang 830054, P.R. China

**Keywords:** phosphodiesterase 4D, single nucleotide polymorphism, cerebral infarction, Uygur

## Abstract

In this study, the correlation between the single nucleotide polymorphisms (SNPs) at rs2910829 and rs918592 in the phosphodiesterase 4D (PDE4D) gene and cerebral infarction in the Uygur and Han ethnic groups of Xinjiang, China were examined. The study population consisted of 373 Uygur and Han patients with cerebral infarction and 377 Uygur and Han control participants with no nervous system diseases. Polymerase chain reaction-restriction fragment length polymorphism (PCR-RFLP) and gene sequencing methods were used to assess the SNPs at the rs2910829 and rs918592 loci in the PDE4D gene. The differences in genotype and allele frequency distribution were compared between the two groups. The C allele frequency of the rs2910829 locus in the PDE4D gene of the cerebral infarction group (81.0%) was significantly higher than that of the control group (76.4%) (P<0.05). Furthermore, the A allele frequency of the rs918592 locus in the PDE4D gene in the Uygur cerebral infarction group was significantly higher than that of the Uygur control group (P<0.05). There were no significant differences in the genotype and allele frequency distributions between the Uygur and Han groups (P>0.05). The A allele of the rs918592 locus may be associated with the occurrence of cerebral infarction in the Uygur population. In addition, it was indicated that the C allele of the rs2910829 locus in the PDE4D gene confers susceptibility to cerebral infarction; however, no significant difference was identified between Uygur and Han patients with cerebral infarction.

## Introduction

Cerebral infarction is a frequently occurring disease of the nervous system, which is a leading cause of mortality and disability worldwide (1). As indicated by pedigree-based studies and case-control studies, the occurrence and development of cerebral infarction exhibit a close correlation with environmental and genetic factors and their interactions (2–4). The phosphodiesterase 4D (PDE4D) gene, a gene of ~1.6 Mb, containing 22 exons and 21 introns, is located on human chromosome 5q12. The encoded protein product is a type of phosphodiesterase, which specifically hydrolyzes cyclic adenosine monophosphate (cAMP) (5). The upregulation of PDE4D expression selectively degrades cAMP, leading to vascular smooth muscle proliferation and migration and an increased local inflammatory response in damaged vessels, thus inducing the development of atherosclerosis and the occurrence of cerebral infarction (6). In 2003, a study by Gretarsdottir *et al*(7) revealed that the PDE4D gene was associated with the occurrence of cerebral infarction in the Icelandic population. A number of similar studies have since been performed; however, the results have not been consistent, possibly due to factors such as individual study backgrounds and ethnic differences in the genetic backgrounds of the populations (8–11).

Xinjiang is a multi-ethnic region in West China, where Uygur and Han are the major ethnic groups, accounting for ~85% of the population. The Uygur population is the largest in Xinjiang. The Uygur people have their own genetic backgrounds and styles of life. Therefore, they are a good natural population for genetic study. The genetic backgrounds and susceptibility genes of the Uygur population are different from those of the Han. In this study, patients with cerebral infarction from the Uygur and Han groups in China were selected and polymorphisms at the rs2910829 and rs918592 gene loci were analyzed. The correlation between the gene polymorphisms and cerebral infarction was investigated in the Uygur and Han groups.

## Patients and methods

### Patients

Among the 373 patients with acute-phase cerebral infarction, 184 were of Uygur ethnicity and 189 were of Han ethnicity (Table I). The diagnoses were performed according to the diagnosing standards set at the Fourth National Cerebrovascular Disease Conference in 1995 (12) and confirmed by cerebral computed tomography (CT) and/or magnetic resonance imaging (MRI) examination. In this study, a further 377 individuals without cerebral infarction served as the control group. Among the 377 participants in the control group, 183 were of Uygur ethnicity and 194 were of Han ethnicity (Table I). The patient data for the individuals in the experimental (cerebral infarction) and control groups are shown in Table I.

All the selected patients were long-term residents in Xinjiang, China, and none of the patients were genetically related. This study was approved by Ethics Committee of Xinjiang Medical University (Urumqi, China), and signed informed consent forms were obtained from all the subjects.

### Polymerase chain reaction (PCR)

Blood samples were collected for the extraction of the DNA templates used in the PCR. The primers used were as follows: rs2910829 forward, 5′-AGGTATGAAGACACCTGAAAGATC-3′ and reverse, 5′-GCAGTATGTTTAAAGATGAGGAAG-3′; rs918592 forward, 5′-CAGAGTGCTGATCAACATTGGT-3′ and reverse, 5′-ATGGAGTCCACAGGGCTTTATT-3′. The primers were synthesized by Sangon Biotech Shanghai Co., Ltd. (Shanghai, China) and thermal cycling amplification was performed using a PCR thermocycler PE2400 instrument (Perkin Elmer, Waltham, MA, USA). Following amplification, the lengths of PCR fragments were 211 and 536 bp for rs2910829 and rs918592, respectively.

### Restriction fragment length polymorphism (RFLP) reaction

The bases of the rs2910829 and rs918592 loci were C, T and A, G, which are able to be digested by restriction enzymes *Ssp*I and *Apa*lI (New England Biolabs, Inc., Ipswich, MA, USA). The PCR products of the rs2910829 and rs918592 loci were sent to Sangon Biotech Shanghai Co., Ltd. and Beijing Biomed Co., Ltd. (Beijing, China) for sequencing. The sequencing results were aligned with the sequences already registered in the Basic Local Alignment Search Tool (BLAST) database [National Center for Biotechnology Information (NCBI); http://blast.ncbi.nlm.nih.gov/] for homologous analysis, in order to confirm the genotypes of the rs2910829 and rs918592 loci determined by digestion.

### Statistical analyses

The results were analyzed using SPSS 17.0 software (SPSS, Inc., Chicago, IL, USA) and are presented as the mean ± standard deviation (SD). Two groups of mean values were compared using an independent samples t-test, while the counting and measurement data were compared using the χ^2^ test. The polymorphic gene frequency distribution was assessed using the Hardy-Weinberg equilibrium test. P<0.05 was considered to indicate a statistically significant difference.

## Results

### Clinical data analysis of patients in the experimental and control groups

To evaluate the correlation between the single nucleotide polymorphisms (SNPs) of the PDE4D gene and the risk of cerebral infarction in the Uygur and Han ethnic groups of Northwest China, 373 patients with acute-phase cerebral infarction and 377 individuals without cerebral infarction were enrolled in this study. The patient data are shown in Table I. There were no statistically significant differences in age, gender or ethnicity between the two groups, suggesting that the experimental and control groups were comparable. The experimental group had significantly higher common risk factors for cerebral infarction, such as hypertension, history of diabetes, hyperlipidemia, history of drinking and body mass index (BMI) compared with the control group (Table I; P<0.05).

### Results of digestion and sequencing for rs2910829 in each group

**Figure 1:**
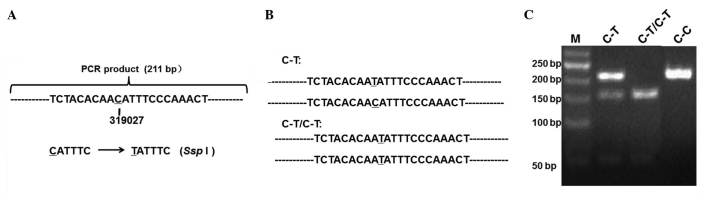
Single nucelotide polymorphisms (SNPs) at the rs2910829 locus of the phosphodiesterase 4D (PDE4D) gene. (A) A putative single-base C-to-T alteration at the rs2910829 locus of the PDE4D gene generated an additional *Ssp*I locus. (B) The single-base C-to-T alteration may occur at the rs2910829 locus of one allele (termed as C–T) or the two alleles (termed as C–T/C–T). (C) SNPs at the rs2910829 locus of the PDE4D gene, as detected using electrophoresis. The experiments were performed ≥3 times. M, DNA marker (50, 100, 150, 200 and 250 bp ladders).

To determine the SNPs at the rs2910829 locus of the PDE4D gene, PCR-RFLP assays were performed. It was observed that the PCR-RFLP experiments generated three types of bands (Fig. 1): One band for the CC genotype, with a fragment length of 211 bp; three bands for the CT genotype, with fragment lengths of 211, 159 and 52 bp; and two bands for the TT genotype, with fragment lengths of 159 and 52 bp. The three types of genotypes following digestion (CC, CT and TT) were consistent with the results of sequencing. The genotype polymorphism distribution of each group conformed to the Hardy-Weinberg equilibrium.

### Results of digestion and sequencing for rs918592 in each group

**Figure 2:**
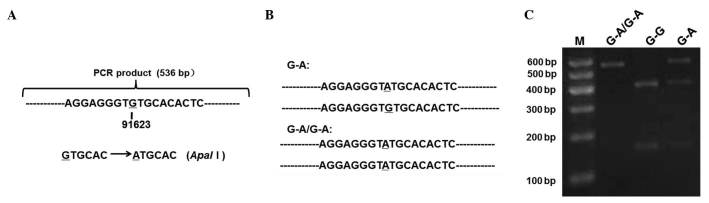
Single nucelotide polymorphisms (SNPs) at the rs918592 locus of the phosphodiesterase 4D (PDE4D) gene. (A) A putative single-base G-to-A alteration at the rs918592 locus of the PDE4D gene generated an additional *Apa*lI locus. (B) The single-base G-to-A alteration may occur at the rs918592 locus of one allele (termed as G–A) or the two alleles (termed as G–A/G–A). (C) SNPs at the rs918592 locus of the PDE4D gene, as detected using electrophoresis. The experiments were performed ≥3 times. M, DNA marker (100, 200, 300, 400, 500 and 600 bp ladders).

To evaluate the SNPs at the rs918592 locus of the PDE4D gene, PCR-RFLP assays were performed. It was observed that the PCR-RFLP experiments generated three types of bands (Fig. 2): One band for the AA genotype, with a fragment length of 536 bp; three bands for the AG genotype, with fragment lengths of 536, 388 and 148 bp; and two bands for the GG genotype, with fragment lengths of 388 and 148 bp. The three genotypes (AA, AG and GG) following digestion were confirmed using DNA sequencing. The genotype polymorphism distribution of each group conformed to the Hardy-Weinberg equilibrium.

### Comparison of the genotype and allele frequency distribution in the rs2910829 and rs918592 loci of the PDE4D gene between the experimental and control groups

To study the correlation between the genotype and allele frequency distributions in the rs2910829 locus and the risk of cerebral infarction, the genotype and allele frequency distribution in the rs2910829 locus was analyzed. The ranking of frequencies for rs2910829 in the PDE4D gene for the experimental and the control group was CC > CT > TT. There was no statistically significant difference in the frequency distribution among the three genotypes (χ^2^=5.765, P=0.056; P>0.05). The C and T allele distributions in the rs2910829 locus in the PDE4D gene of the experimental group were 81.0 and 19.0%, respectively (Table II). The C and T allele distributions of the control group were 76.4 and 23.6%, respectively. The C allele distribution of the experimental group was significantly higher than that of the control group (χ^2^=4.672, P=0.031; P<0.05). The individuals with C alleles had a higher risk of cerebral infarction than those with T alleles, with an odds ratio (OR) value of 1.314 (95% CI, 1.025–1.685; P<0.05; Table II).

The ranking of frequencies for the rs918592 locus in PDE4D gene of the experimental and the control group was AG > GG > AA. There was no statistically significant difference in the frequency distribution among the three genotypes (χ^2^=2.910, P=0.233; P>0.05). There was also no statistically significant difference in the frequency distributions for the A and G alleles (χ^2^=0.002, P=0.965; P>0.05; Table III).

These results suggested that the C allele in the rs2910829 locus of the PDE4D gene may indicate susceptibility to cerebral infarction.

### Comparison of genotype and allele frequency distributions of the rs2910829 and rs918592 loci in the PDE4D gene between the Uygur and Han ethnic groups in the experimental and control groups

The comparison of the rs2910829 locus between the Uygur and Han ethnic groups of Northwest China indicated that the CC genotype and the C allele frequencies of the rs2910829 locus in the PDE4D gene of the Uygur individuals in the experimental group were higher than those of the Uygur individuals in the control group, although there was no statistically significant difference in the C allele frequency distribution (χ^2^=2.534, P=0.11; P>0.05; Table IV). The CC genotype and C allele frequencies at the rs2910829 locus in the PDE4D gene of the Han ethnic experimental group were higher than those of the Han ethnic control group, although there was no statistically significant difference in the C allele frequency distribution (χ^2^=2.254, P=0.133; P>0.05). Similarly, there were no statistically significant differences in the genotype and allele frequency distributions in the Uygur and Han ethnic groups (P>0.05).

The comparison of the rs918592 locus in the Uygur and Han ethnic groups of Northwest China indicated that the AA genotype and A allele frequencies of the rs918592 locus in the PDE4D gene of the Uygur ethnic experimental group were higher than those of the Uygur ethnic control group, and there was a statistically significant difference in the A allele frequency distribution (χ^2^=4.326, P=0.038; P<0.05; Table V). The Uygur individuals with A alleles had a higher risk of cerebral infarction than those with G alleles, with an OR value of 1.044 (95% CI, 1.026–1.062; P<0.05). There were no statistical differences in the genotype and allele frequency distributions between the Han individuals in the experimental group and those in the control group (P>0.05). In addition, the frequency distributions of the three genotypes of the rs918592 locus in the PDE4D gene showed no statistically significant differences between the Uygur and Han ethnic groups (χ^2^=0.466, P=0.792; P>0.05). There were also no statistical differences in the frequency distributions of the A and G alleles (χ^2^=0.525, P=0.469; P>0.05). These results suggested that there was no significant difference in the rs2910829 polymorphisms of the PDE4D gene between the Uygur and Han ethnic groups. The polymorphism in the rs918592 locus may be associated with the occurrence of cerebral infarction in the Uygur group. The individuals carrying an A allele may have a higher risk of cerebral infarction than those carrying a G allele in this locus.

## Discussion

The Uygur population in Xinjiang, Northwest China, has a relatively unique life style and genetic background. The distinct genetic background of the Uygur population from the Han population may lead to differences in genes conferring genetic susceptibility. In this study, the genotype polymorphisms of the rs2910829 and rs918592 loci in the PDE4D gene of the Uygur and Han populations were evaluated. The results showed that the CC genotype of the rs2910829 locus of the PDE4D gene in Uygur and Han patients with cerebral infarction had the highest distribution frequency, while the frequency distribution of the C allele was higher than that of the T allele. During the investigation into the correlation between the polymorphisms of the rs2910829 locus and cerebral infarction, it was observed that the frequency of the C allele in the experimental group was significantly higher than that in the control group (P<0.05). It was indicated that individuals with C alleles were at a higher risk of cerebral infarction than those with T alleles (OR value of 1.314), which was consistent with the results of a study of the Han population in Harbin by Zhang *et al*(13). The CC genotype and C allele frequencies of the rs2910829 locus for the Uygur and Han populations in the experimental group were higher than those in the control group, although there were no statistically significant differences. Similarly, the genotype and allele frequency distributions of the rs2910829 locus showed no statistically significant differences between the Uygur and Han populations (P>0.05). However, it was suggested that the C allele of the rs2910829 locus of the PDE4D gene may be a common allele indicating susceptibility for cerebral infarction in the Uygur and Han populations. It was also suggested that the polymorphisms of the rs2910829 locus may affect the PDE4D gene level and activity changes, alter the intracellular cAMP level and thus promote the formation of atherosclerosis, which is closely associated with the occurrence of cerebral infarction.

The results of the present study showed that the genotype of the rs918592 locus exhibited polymorphisms in the Uygur and Han populations. The AG genotype of the rs918592 locus of the PDE4D gene in the Uygur and Han patients with cerebral infarction had the highest distribution frequency, which was consistent with the results from previous studies (14,15). During the investigation into the correlation between the polymorphisms of the rs918592 locus and cerebral infarction, it was revealed that the genotype and allele frequencies in the experimental group were not statistically different from those of the control group (P>0.05), which was consistent with the conclusions by Xu *et al*(15) and Tang (16). By contrast, Song *et al*(14) discovered that rs918592 was a locus implicated in early-onset strokes in African-American and Caucasian-American females, and was significantly correlated with aortic atherosclerotic cerebral infarction, occlusive cerebral infarction of the small blood vessels and unexplained cerebral infarction. In 2013, He *et al*(17) revealed that the rs918592 and rs2910829 loci were significantly correlated with cerebral infarction in a young Chinese population. In the present respective analyses of the Uygur and Han ethnic groups, it was revealed that there was a statistical difference in the A allele frequency distribution between the experimental and control groups of the Uygur population (P<0.05), suggesting that the polymorphism in the rs918592 locus may be associated with the occurrence of cerebral infarction, and that the individuals with A alleles may have a higher risk of cerebral infarction than the individuals with G alleles. Due to the small number of the individuals in the samples, this result may be unreliable. Future studies with larger sample sizes are required, in order to further verify this result.

Since 2003, when Gretarsdottir *et al*(7) demonstrated that the PDE4D gene was a novel risk factor for cerebral infarction, studies concerning the correlation between PDE4D gene polymorphisms and cerebral infarction have made certain progress; however, disputes remain. A number of studies have suggested that the PDE4D gene is correlated with cerebral infarction in Asian (18), European (19) and North American (20) populations; however, other studies have indicated that these correlations do not exist. In the USA, Woo *et al*(20) studied Caucasian- and African-Americans and showed that SNP45 of the PDE4D gene exhibited no polymorphism, whereas SNP41 was associated with cardioembolic stroke in Caucasian-Americans. Lövkvist *et al*(9) performed a multi-center study involving a large sample of 2,599 patients with ischemic stroke and 2,093 control subjects from the south and west regions of Sweden and did not identify any correlation between the examined subgroups and SNP45 with ischemic stroke. Munshi *et al*(21) demonstrated a significant correlation of SNP56 and SNP41 with large artery atherosclerosis, and lacunar and cardioembolic stroke in the South Indian population from Andhra Pradesh. Furthermore, Matsushita *et al*(10) studied 5 SNPs in the Japanese population; however, no correlation was revealed between PDE4D gene polymorphisms and ischemic stroke. Bevan *et al*(11) performed a meta-analysis of the PDE4D gene in 5,200 cases and 6,600 controls, and suggested that the PDE4D gene was not associated with cerebral infarction and that the correlation in previous studies was limited to certain specific populations or subgroups. By contrast, Xu *et al*(22) performed a meta-analysis to investigate the correlation between the PDE4D gene and cerebral infarction in Asian patients between 1984 and 2009, and suggested that only SNP83 was notably correlated with cerebral infarction. There were also certain differences in the results of the present study, for which the reasons may be: i) Cerebral infarction is a complex disease with multiple causative factors and is dependent on environmental and genetic factors, whereas the Uygur population in Xinjiang may have unique habits and customs that affect the occurrence of cerebral infarction; ii) there are differences in the genetic factors of different ethnic groups; iii) different study methods, inclusion and exclusion criteria and sample sizes were used.

In this study, case-control and molecular biology methods were combined to investigate the PDE4D gene in the patients with cerebral infarction in Xinjiang. The results indicated that the rs2910829 and rs918592 loci of the PDE4D gene of Uygur and Han ethnic groups in Xinjiang exhibited polymorphic distributions. Following the analysis of the correlation of the rs2910829 and rs918592 loci of the PDE4D gene with cerebral infarction in the Uygur and Han populations in this region, it was identified that the C allele at the rs2910829 locus of the PDE4D gene may be a common allele indicating susceptibility to cerebral infarction. However, there was no difference between the Uygur and Han patients with cerebral infarction. Similarly, the A allele of the rs918592 locus may be correlated with the occurrence of cerebral infarction in Uygur individuals.

## Figures and Tables

**Table I:** Comparison of data between the experimental (cerebral infarction) and control groups.

Variable	Experimental group (n=226)	Control group (n=220)	T-value or χ^2^ value	P-value
Age (years, mean ± SD)	59.86±13.18	58.81±10.11	1.219^a^	0.223
Gender (m/f)	246/127	240/137	0.432	0.511
Ethnic group (Han/Uygur)	189/184	184/183	0.470	0.829
Body mass index (normal/abnormal)	201/172	87/290	75.244	0.000^b^
History of smoking (yes/no)	121/252	54/323	34.398	0.000^b^
History of drinking (yes/no)	80/293	25/352	34.186	0.000^b^
History of hypertension (yes/no)	232/141	66/311	156.391	0.000^b^
History of diabetes (yes/no)	111/262	33/344	53.568	0.000^b^
Hyperlipidemia (yes/no)	94/279	23/354	51.952	0.000^b^

a T-value;

b P<0.05, comparison with the control group.

**Table II:** Comparison of genotype and allele frequency distributions of the PDE4D gene rs2910829 locus between the experimental (cerebral infarction) and control groups.

		Genotype frequency (%)			Allele frequency (%)	
			
Group	n	TT	CT	CC	T	C
Experimental	373	19 (5.1)	104 (27.9)	250 (67.0)	142 (19.0)	604 (81.0)^a^
Control	377	22 (5.8)	134 (35.5)	221 (58.6)	178 (23.6)	576 (76.4)

a Comparison with the control group, P<0.05.

PDE4D, phosphodiesterase 4D.

**Table III:** Comparison of genotype and allele frequency distribution of the PDE4D gene rs918592 locus between the experimental (cerebral infarction) and control groups.

		Genotype frequency (%)			Allele frequency (%)	
			
Group	n	AA	AG	GG	A	G
Experimental	373	101 (27.1)	157 (42.1)	115 (30.8)	359 (48.1)	387 (51.9)
Control	377	90 (23.9)	182 (48.3)	105 (27.9)	362 (48.0)	392 (52.0)

PDE4D, phosphodiesterase 4D.

**Table IV:** Comparison of genotype and allele frequency distributions of the PDE4D gene rs2910829 locus between the Uygur and Han ethnic groups.

			Genotype frequency (%)			Allele frequency (%)	
				
Group	Ethnic group	n	TT	CT	CC	T	C
Experimental	Uygur	184	12 (6.5)	56 (30.4)	116 (63.0)	80 (21.7)	288 (78.3)
	Han	189	7 (3.7)	48 (25.4)	134 (70.9)	62 (16.4)	316 (83.6)
Control	Uygur	183	14 (7.7)	70 (38.3)	99 (54.1)	98 (26.8)	268 (73.2)
	Han	194	8 (4.1)	64 (33.0)	122 (62.9)	80 (20.6)	308 (79.4)

PDE4D, phosphodiesterase 4D; experimental group, patients with cerebral infarction.

**Table V:** Comparison of genotype and allele frequency distributions of the PDE4D gene rs918592 locus between the Uygur and Han ethnic groups.

			Genotype frequency (%)			Allele frequency (%)	
				
Group	Ethnic group	n	AA	AG	GG	A	G
Experimental	Uygur	184	53 (28.8)	77 (41.8)	54 (29.3)	183 (49.7)^a^	185 (50.3)
	Han	189	59 (31.2)	80 (42.3)	50 (26.5)	198 (52.4)	180 (47.6)
Control	Uygur	183	34 (18.6)	86 (47.0)	63 (34.4)	154 (42.1)	212 (57.9)
	Han	194	56 (28.9)	96 (49.5)	42 (21.6)	208 (53.6)	180 (46.4)

a P<0.05 in comparison with Uygur in the control group.

PDE4D, phosphodiesterase 4D; experimental group, patients with cerebral infarction.
